# Clinical Management and Outcome of Grade III Pneumonitis after Chemoradioimmunotherapy for Inoperable Stage III Non-Small Cell Lung Cancer—A Prospective Longitudinal Assessment

**DOI:** 10.3390/diagnostics11111968

**Published:** 2021-10-23

**Authors:** Diego Kauffmann-Guerrero, Julian Taugner, Chukwuka Eze, Lukas Käsmann, Minglun Li, Amanda Tufman, Farkhad Manapov

**Affiliations:** 1 Divison of Respiratory Medicine and Thoracic Oncology, Department of Internal Medicine V and Thoracic Oncology Centre Munich (TOM), Hospital of the University of Munich (LMU), 80336 Munich, Germany; Amanda.Tufman@med.uni-muenchen.de; 2 Member of the German Center for Lung Research (DZL), Center for Lung Research (DZL), Comprehensive Pneumology Center Munich (CPC-M), 81377 Munich, Germany; Lukas.Kaesmann@med.uni-muenchen.de (L.K.); Minglun.Li@med.uni-muenchen.de (M.L.); Farkhad.Manapov@med.uni-muenchen.de (F.M.); 3 Department of Radiation Oncology, University Hospital LMU, 80336 Munich, Germany; Julian.Taugner@med.uni-muenchen.de (J.T.); Chukwuka.Eze@med.uni-muenchen.de (C.E.); 4 German Cancer Consortium (DKTK), Partner Site Munich, 80336 Munich, Germany

**Keywords:** chemoradioimmunotherapy, NSCLC, pneumonitis, lung function, immunotherapy

## Abstract

Background: Maintenance treatment with immune-checkpoint inhibition (ICI) has been shown to significantly improve patient prognosis after chemoradiotherapy (CRT) for inoperable stage III NSCLC. This survival advantage may be achieved at the expense of an increased probability for symptomatic pneumonitis as CRT as well as ICI treatment is associated with the risk of treatment-related pulmonary toxicity. Methods: We screened a prospective chemoradioimmunotherapy (CRT-IO) cohort consisting of 38 patients and identified patients with therapy-related grade 3 pneumonitis. All patients were treated with intravenous high dose corticosteroids and closely monitored by CT-scans and extended longitudinal lung function tests. We analyzed lung function parameters and CT morphological features to characterize patients’ outcome. Results: Six (16%) patients treated with CRT-IO developed grade 3 pneumonitis one to six months after completion CRT. In the CT imaging, pneumonitis was characterized by diffuse ground glass capacities and in part pulmonary consolidations within and outside the planning target volume. Onset of pneumonitis was accompanied by a reduction in diffusion capacity in all cases. The mean decline of diffusion capacity was 25.8% [6–53%]. Under treatment with corticosteroids, all patients recovered regarding symptoms and changes in CT morphology. In five out of six patients, diffusion capacity improved to at least 80% of the baseline [80–96%]. One patient showed a significant increase of diffusion capacity after treatment (from 32% to 53%) but reached only 62% of the initial value. Conclusions: Pneumonitis is a severe complication of CRT-IO. High-resolution CT imaging and extended lung function testing proved to be a suitable approach in detecting and monitoring of CRT-IO associated pneumonitis.

## 1. Introduction

Non-small cell lung cancer (NSCLC) is the leading cause of cancer-related death worldwide [1]. Despite continuous progress in systemic therapy, treatment of patients with metastatic and/or locally advanced disease remains challenging. Standard of care for patients with locally advanced NSCLC ineligible for surgery is chemoradiotherapy (CRT) [2]. Additionally, concomitant multimodal treatment leads to significantly improved patient outcome compared to sequential protocols [2].

However, local and distant relapse is a common event occurring in at least two thirds of CRT patients [3]. The last years have shown that maintenance therapy with immune checkpoint inhibitors (ICIs) could significantly improve progression-free (PFS) and overall survival (OS) in advanced NSCLC. Therefore, combination of chemotherapy and immunotherapy is now the standard of care in the first-line treatment of metastatic disease [4,5,6]. Furthermore, it has been shown that the combination of radiotherapy and ICIs provides synergistic effects [7]. This was corroborated by the findings of the PACIFIC trial in which consolidation with the PD-L1 inhibitor durvalumab after CRT significantly prolonged PFS and OS in patients with inoperable stage III NSCLC [8,9,10]. Corresponding real-world studies confirmed the PACIFIC results and reported one-year PFS rates in the order of 50–65% [9,11,12]. In addition, combinations of CRT with different anti-PD-1 and anti-PD-L1 drugs are currently under evaluation [13,14,15,16].

Advances in patient survival after implementation of ICIs are often associated with treatment-related toxicity. Especially pneumonitis, which is a common and sometimes severe adverse event of both, thoracic irradiation (TRT) and immune checkpoint inhibition, may be initiated by combining these modalities [3,7,8]. Irradiation induces cellular mechanisms leading to alveolar inflammation by pro-inflammatory cytokines and chemokines (e.g., TGF-β, PDGF and/or interleukins) and reactive oxygen species [17]. Release of cellular immune response by concomitant or sequential PD-L1 blockade, may intensify these reactions leading to a higher incidence and severity.

Effective strategies for early detection and consequent treatment algorithms are essential for successful management of pneumonitis. Herein, we describe a prospective single-center experience of clinical management and outcomes of severe pneumonitis after CRT combined with ICI.

## 2. Materials and Methods

Six (16%) of 38 patients with inoperable stage III NSCLC receiving CRT combined with ICI at our tertiary cancer center developed grade 3 pneumonitis. There was no grade 4–5 pneumonitis in the treated cohort. We used the current version of the CTCAE (Common Terminology Criteria for Adverse Events) classification to grade pneumonitis. Grade 3 is defined as severe symptoms leading to limited selfcare, need of additional oxygen and cortisone treatment. Patient- and treatment characteristics, longitudinal diagnostic imaging and pulmonary function tests were prospectively collected and analyzed. All six patients were initially treated with high dose intravenous prednisolone (1.5–2 mg/kg body weight).

Lung function parameters were calculated according the Global Lung Function Initiative (GLI) of the European Respiratory Society (ERS) [18]. We correlated vital capacity (VCmax), forced expiratory volume in one second (FEV1) and diffusion capacity (corrected for alveolar volume and hemoglobin, (DLCOc/VA)) with imaging and response to prednisolone treatment.

Four (67%) patients received durvalumab maintenance after concurrent CRT. Two (33%) patients were treated in the phase II NICOLAS trial [15] and received concomitant chemoradioimmunotherapy followed by a nivolumab consolidation. Table 1 summarizes patient characteristics and treatment modalities.

All patients provided signed informed consent. The study was approved by the Institutional Review Board of the University hospital of the Ludwig-Maximilians-University (approval number: 17–230) and was conducted according to local and federal regulations and the Declaration of Helsinki.

## 3. Results

### 3.1. Patient 1

**Figure 1:**
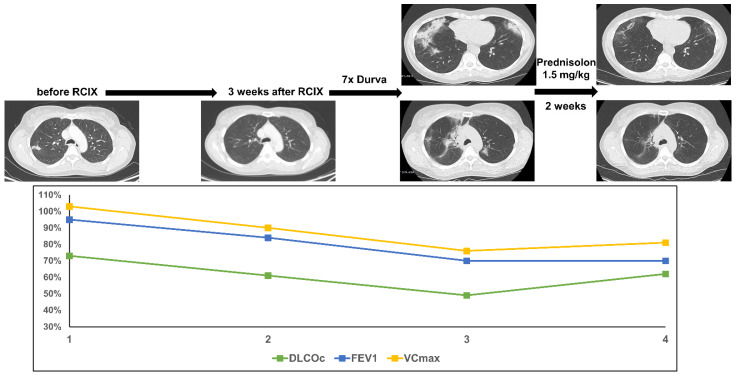
The figure shows course of treatment, corresponding CT imaging and lung function values of patient 1. (RCIX: Radiochemoimmunotherapy; Durva: Durvalumab).

This woman was diagnosed with an adenocarcinoma of the lung at the age of 47. The disease presented with a small pulmonary tumor, but extensive mediastinal and cervicallymph node metastases (cT3 cN3 cM0). The patient was treated with 4 cycles of neoadjuvant chemotherapy with cisplatin and pemetrexed and demonstrated a partial response. Concomitant CRT followed (PTV 858.9cc, V20 total lung 27.5% and mean lung dose 14.51 Gy). As the tumor cells revealed a PD-L1 expression of 80%, consolidation with durvalumab was initiated 3 weeks after completion of CRT. After the seventh cycle of durvalumab [6 months after end of thoracic irradiation (TRT)] the patient presented with acute dyspnea CTC grade 2. A lung function test showed a decline of DLCOc/VA by 24% accompanied by a reduced VCmax and FEV1. Computer tomography (CT) scanning showed new bilateral ground glass opacities with beginning consolidations. After exclusion of malignancy and analysis of lymphocytic bronchoalveolar lavage (BAL) the patient was treated with oral high-dose prednisolone (1.5 mg/kg body weight). Within two weeks, near-complete resolution of dyspnea and ground glass opacities occurred. DLCOc/VA recovered to the initial value, but decline in VCmax and FEV1 persisted as a surrogate for post-therapeutic fibrosis (Figure 1).

### 3.2. Patient 2

**Figure 2:**
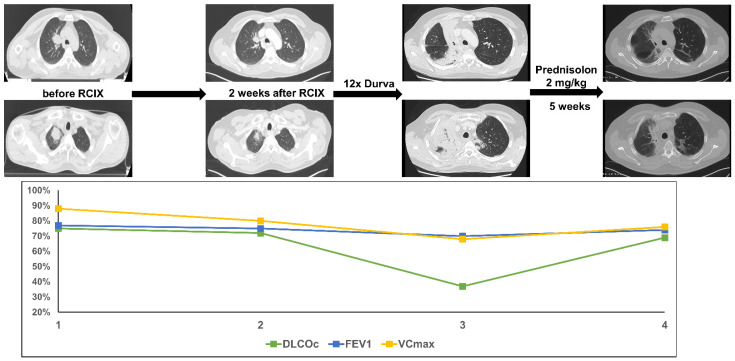
The figure shows course of treatment, corresponding CT imaging and lung function values of patient 2. (RCIX: Radiochemoimmunotherapy; Durva: Durvalumab).

A male patient was diagnosed with squamous cell carcinoma of the lung at the age of 51. The disease presented with two pulmonary tumors in the right upper lobe and ipsilateral paratracheal lymph node metastases (cT3 cN2 cM0). Concomitant CRT with cisplatin and vinorelbine was initiated (PTV 847.2cc, V20 total lung 14.7% and mean lung dose 9.27 Gy). As the tumor cells revealed a PD-L1 expression of 10%, consolidation with durvalumab was started 2 weeks after completion of CRT. After twelve cycles of durvalumab (3.5 months post-TRT) the patient presented with acute dyspnea CTC grade 3. Lung function testing revealed a decline in DLCOc/VA by 38% accompanied by only a slight decline in VCmax and FEV1. CT scanning showed extensive consolidations in the right upper lobe. The patient was treated with high-dose oral prednisolone (2 mg/kg body weight). Few days after initiation of steroid treatment, the dyspnea improved substantially. Five weeks later, a CT scan showed near-complete resolution of the consolidations and residual fibrosis. DLCOc/VA improved to the initial value and as in patient 1, decline in VCmax and FEV1 persisted as an expression of post-therapeutic fibrosis (Figure 2).

### 3.3. Patient 3

**Figure 3:**
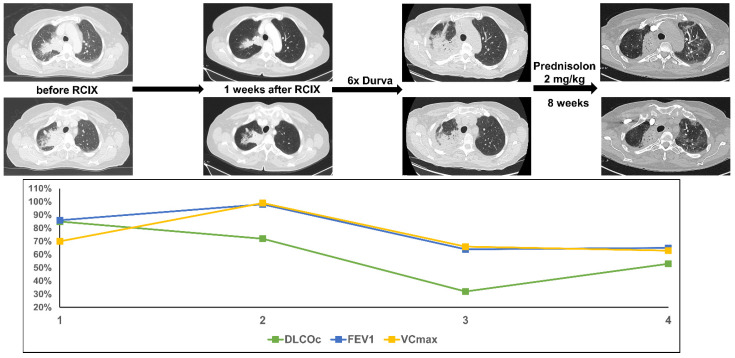
The figure shows course of treatment, corresponding CT imaging and lung function values of patient 3. (RCIX: Radiochemoimmunotherapy; Durva: Durvalumab).

This woman was diagnosed with an adenocarcinoma of the lung at the age of 72 and presented with a large pulmonary tumor in the right upper lobe and ipsilateral hilar lymph node metastases (cT4 cN2 cM0). The patient was treated with 2 cycles of neoadjuvant chemotherapy with cisplatin and pemetrexed and re-evaluated for curative-intent surgery. After induction chemotherapy, stable disease was achieved thus concomitant CRT with carboplatin and vinorelbine was started (PTV 1135.0cc, V20 total lung 25.3% and mean lung dose 14.27 Gy). After six cycles of durvalumab (4 months post-TRT) the patient presented with acute dyspnea CTC grade 3 and productive cough. Lung function testing showed a decline in diffusion capacity by 53% accompanied by a reduced vital capacity and FEV1. As in patient 2, CT scanning showed new extensive consolidations in the right upper lobe with surrounding ground glass opacities. The patient was treated with high-dose i.v. and oral prednisolone (2 mg/kg body weight). Few days after the beginning of steroid treatment, the dyspnea improved significantly. The consolidations and ground glass opacities were dramatically regredient after 5 weeks of cortisone treatment, but a large fibrotic area remained. DLCOc/VA improved by 21%, but VCmax and FEV1 reduction persisted as an expression of post-therapeutic fibrosis (Figure 3).

### 3.4. Patient 4

**Figure 4:**
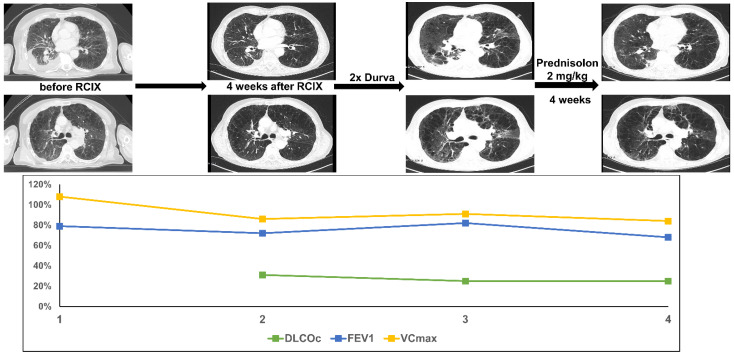
The figure shows course of treatment, corresponding CT imaging and lung function values of patient 4. (RCIX: Radiochemoimmunotherapy; Durva: Durvalumab).

A 70-year-old patient was diagnosed with cT2a cN0 cM0 squamous cell carcinoma in the right lower lobe. A sublobar resection was performed. Only 2 months later, the patient was re-admitted with a new left hilar tumor and lymph node metastases (rcT2 rcN2 cM0). Concomitant CRT with cisplatin and vinorelbine was initiated (PTV 675.6cc, V20 total lung 29.0%, and mean lung dose 14.26 Gy). As the tumor cells revealed a PD-L1 expression of 60%, consolidation with durvalumab was initiated 4 weeks after the completion of CRT. Just after two cycles of durvalumab (2 months after end of thoracic irradiation) the patient presented with acute dyspnea CTC Grade 3. Lung function testing revealed a mild decrease in diffusion capacity by 6%, which represented a fifth of the baseline value. CT scanning showed new bilateral ground glass opacities. The patient was treated with high-dose i.v. and oral prednisolone (2 mg/kg body weight). Shortly after initiation of cortisone treatment, the dyspnea improved. 4 weeks later, a CT scan showed significantly regredient ground glass opacities. There was no improvement in lung function values, probably because the lung function was already quite impaired before the start of multimodal treatment (Figure 4).

### 3.5. Patient 5

**Figure 5:**
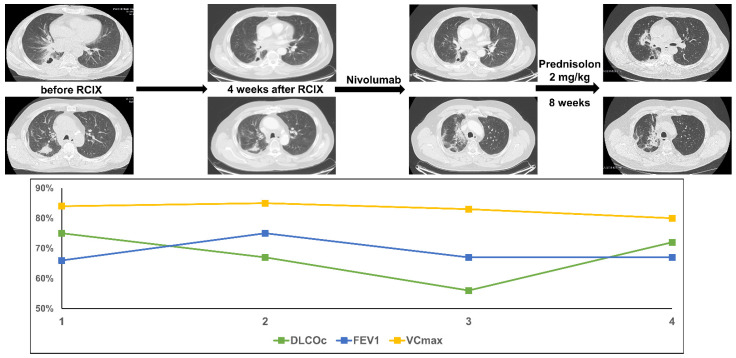
The figure shows course of treatment, corresponding CT imaging and lung function values of patient 5. (RCIX: Radiochemoimmunotherapy).

This male patient was diagnosed with squamous cell carcinoma of the lung at the age of 58 and presented with a central pulmonary tumor, a pulmonary satellite tumor in the right upper lobe and N3 mediastinal lymph node metastases (cT4 cN3 cM0). Concomitant CRT with cisplatin, vinorelbine and nivolumab was initiated within the NICOLAS trial (PTV 1189.2cc, V20 total lung 25.4% and mean lung dose 15.41 Gy). The tumor cells showed no PD-L1 expression. As specified by the study protocol, consolidation therapy with nivolumab was started. Two months later and after 4 cycles of nivolumab consolidation, the patient presented with progressive dyspnea CTC Grade 2 and dry cough. Lung function testing revealed a decline in DLCOc/VA by 19% accompanied by only a slight reduction of FEV1. CT scanning showed new fibrotic lesions and diffuse ground glass opacities in the right upper lobe. The patient was treated with high-dose oral prednisolone (2 mg/kg body weight). Shortly after initiation of steroid treatment, the dyspnea improved. 8 weeks later a CT scan confirmed a significant reduction of ground glass opacites but showed residual fibrotic areas. DLCOc/VA recovered to the initial value and VCmax and FEV1 stayed slightly reduced as an expression of post-therapeutic fibrosis (Figure 5).

### 3.6. Patient 6

**Figure 6:**
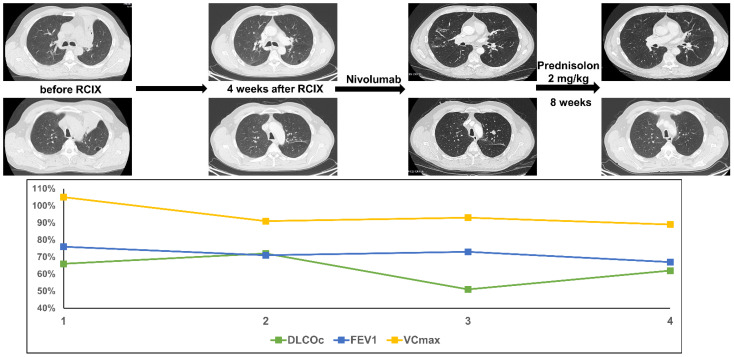
The figure shows course of treatment, corresponding CT imaging and lung function values of patient 6. (RCIX: Radiochemoimmunotherapy).

Patient 6 was diagnosed with squamous cell carcinoma of the lung at the age of 49. The patient presented with a central pulmonary tumor in the left upper lobe with tumor-associated atelectasis and extensive mediastinal lymph node metastases (cT4 cN3 cM0). Concomitant chemoradioimmunotherapy with cisplatin, vinorelbine and nivolumab was initiated within the NICOLAS trail (PTV 867.4cc, V20 total lung 28.4% and mean lung dose 14.75 Gy)**.** As specified by the study protocol, consolidation therapy with nivolumab was started. Four months later, the patient presented with progressive dyspnea CTC grade 3 and dry cough. Lung function testing revealed a decline in DLCOc/VA by 15%. A CT scan showed new bilateral diffuse ground glass opacities. The patient was treated with high-dose oral prednisolone (2 mg/kg body weight). Shortly after initiation of steroid treatment, the dyspnea improved and 8 weeks later a CT scan confirmed a significant reduction of ground glass opacities. DLCOc/VA almost recovered to the baseline value (Figure 6).

## 4. Discussion

Pneumonitis is a common, potentially severe and life-threatening side effect after treatment with chemotherapy, TRT and ICI. Up to 30% of irradiated patients with advanced thoracic malignancies maydevelop radiation-induced symptomatic pneumonitis (RIP) [17]. The incidence of symptomatic ICI-related pneumonitis in monotherapy or chemoimmunotherapy combination studies is lower and ranges from 1–10% [19,20,21]. Combining checkpoint inhibition with concurrent CRT has been revealed to significantly improve survival in locally-advanced NSCLC patients at the expense of a potential increase in the risk of severe treatment-related side effects, including pneumonitis [8,22,23,24]. In the PACIFIC trial, the rate of any-grade pneumonitis was increased in the durvalumab compared to the placebo group (33.0% vs. 24.8%). The incidence of severe grade 3–4 pneumonitis was also higher in the durvalumab (3.4% vs. 2.6%) compared to standard arm [8]. Real-world studies from Shaverdian et al. and Jung et al. have reported on significant higher rates of symptomatic Grade II-III pneumonitis in the patients treated with concurrent CRT followed by durvalumab [9,25]. In another prospective study of 26 inoperable stage III NSCLC patients treated with durvalumab maintenance, an 18% rate of grade III pneumonitis was reported [11].

Herein, we present a prospective longitudinal experience of six cases with Grade III pneumonitis after treatment with concurrent CRT and ICI. All cases shared a similar pattern and clinical parameters of this adverse event, which might help to establish a sensitive surveillance and treatment algorithm.

In all patients, symptomatic pneumonitis developed 1–6 months after completion of concurrent CRT and during consolidation treatment with ICI. This is in line with recent studies showing, that pneumonitis after this tri-modal approach occurred within a median time of 3.4 months after end of TRT [25]. Symptoms were acute, in part significant dyspnea CTC grade 2–3 and coughing. All patients were at least former smokers, which might be an additional risk factor. CT morphological correlates were diffuse ground glass opacities and interstitial edema and pulmonary consolidations within and outside the planning target volume. This also correlates with previously published data of radiation- and ICI-related pneumonitis. The radiological pattern of ICI-related pneumonitis is very heterogeneous and ranges from diffuse ground glass opacities to cryptogenic organizing pneumonia and different patterns of fibrosis [17,20,26].

Only one patient (patient 1) in our cohort underwent bronchoscopy with BAL to exclude infection and confirm lymphocytic alveolitis. All other patients did not receive bronchoscopic examination due to their reduced respiratory condition. However, we recommend bronchoscopy with BAL whenever possible in these patients.

In our cohort, a decline in diffusion capacity (DLCOc/VA) proved a sensitive pneumonitis-associated parameter with correlation to the onset and severity of dyspnea. We found a mean decline in diffusion capacity of 25.8% [range 6–53%]. Furthermore, the extent of ground glass opacities and consolidations correlates well with the decrease in DLCOc/VA. Importantly, in 5 (83%) patients, the DLCOc/VA recovered to at least 80% of the baseline values [range 80–96%] after treatment with corticosteroids. One patient showed a significant increase of diffusion capacity after treatment (from 32% to 53%) but plateaued at only 62% of the initial value. This patient had a pre-existing limited lung function and diffusion capacity (COPD GOLD II) before initiation of TRT. Additionally, concurrent CRT followed by durvalumab was started only 2 months after the patient had sublobar resection of a contralateral pulmonary tumor. Both conditions might be additional risk factors for the development and severity of pneumonitis. Morimoto and colleagues recently published a study on the role of COPD in the application of immunotherapy after completion of concurrent CRT [27].

The mean decline of DLCOc/VA from baseline in the group of the 32 patients who did not develop severe pneumonitis was 13.3% three months after CRT, 8.6% six months after CRT and 7.1% twelve months after CRT. In contrast the mean of maximal DLCOc/VA decline in the six patients reported in our manuscript was 25.8%. 

Therefore, DLCOc/VA might be considered as an easy to obtain parameter to continuously monitor inoperable stage III patients during and after completion of tri-modal therapy. Furthermore, DLCOc/VA can be used to evaluate both onset of pneumonitis and response of therapy-related pneumonitis to corticosteroid treatment. VCmax and FEV1 did not correlate with the severity of pneumonitis in our study. These parameters rather serve as surrogates for the extent of irreversible lung tissue damage after multimodality treatment including chemotherapy, TRT and ICI.

High-dose prednisolone (1.5–2 mg/kg body weight) was administered to treat severe pneumonitis and rapid clinical improvement within a few days was observed. Symptom-oriented tapering of corticosteroids, longitudinal CT scans and lung function testing were revealed as promising management strategies. In our approach, we use a higher initial dose of corticosteroids than used in other reports [28]. Naidoo and colleagues recently published recommendations for the treatment of pneumonitis after concurrent CRT followed by ICI [3]. The authors recommend treating severe pneumonitis with 1–2 mg/kg body weight for a few days and symptom- and imaging-driven corticosteroid tapering strategies. This is in close accordance with our treatment algorithm. After initial intravenous administration of prednisolone (1.5–2 mg/kg body weight), we switched early to oral administration after relief of severe symptoms. Steroid doses were slowly tapered over several weeks guided by patients’ symptoms, lung function and imaging. 

Additionally, we found that DCLOc/VA seems to be a sensitive parameter for screening and follow-up of treatment-related pneumonitis in this group of patients. Therefore, we recommend continuous lung function testing including measurement of diffusion capacity to be included in the surveillance and follow-up program of inoperable stage III NSCLC patients receiving CRT combined with ICI.

In our study ICI consolidation was interrupted by onset of symptomatic pneumonitis in all patients. In addition, there was no ICI re-challenge after completion of pneumonitis therapy. There is recent data showing that interruption or discontinuation of ICI due to side effects does not affect patient outcome or recurrence rate in this group of inoperable stage III NSCLC [24].

Although this is a small single-center longitudinal study, clinical practice showed that symptomatic pneumonitis in the course of tri-modal treatment for inoperable stage III NSCLC is a relevant event. Additionally, timing and pattern of care for symptomatic pneumonitis is in close accordance with recently published data. Proactive and structured longitudinal approach, including symptom control (dyspnea and cough), lung function testing with DLCO and CT imaging are obligatory to provide an optimal patient-centered care. Rapid initiation of high-dose cortisone therapy is pertinent in the management of symptomatic pneumonitis and leads to rapid recovery in the absolute majority of patients.

## 5. Conclusions

In this report, we present a prospective single-center longitudinal experience of six cases with grade III pneumonitis in inoperable stage III NSCLC patients after treatment with concurrent CRT and ICI. Severe pneumonitis is a life-threatening complication of CRT-IO. High-dose steroid treatment rapidly relieved patients’ symptoms and improved lung function parameters. Close and longitudinal monitoring with CT and extended lung function testing including DLCO revealed to be an effective approach to detect and timely treat this severe complication.

## Figures and Tables

**Table 1:** **The table shows patients baseline characteristics and treatment modalities.** PD-L1 (Programmed Death Ligand 1, ≥1% indicates a positive case with an expression of lower than 5%), ECOG (Eastern Cooperative Oncology Group).

	Patient 1	Patient 2	Patient 3	Patient 4	Patient 5	Patient 6
**Gender**	female	male	female	male	male	male
**Age at diagnosis**	47	51	72	70	58	49
**Histology**	Adenocarcinoma	Squamous cell carcinoma	Adenocarcinoma	Squamous cell carcinoma	Squamous cell carcinoma	Squamous cell carcinoma
**PD-L1**	80%	10%	≥1%	60%	0%	≥1%
**Tumor stage**	cT3 cN3 cM0 (UICC IIIC)	cT3 cN2 cM0 (UICC IIIB)	cT4 pN2 cM0 (UICC IIIB)	Initial pT2a N0 M0 N3-relaps, (UICC IIIB)	cT4 cN3 cM0 (UICC IIIC)	cT4 cN3 cM0 (UICC IIIC)
**ECOG staus at diagnosis**	0	1	1	2	0	0
**Smoking status**	Former (20 py)	Former (30 py)	Former (30 py)	Former (50 py)	Former (27 py)	Active (35 py)
**Irradiation protocol**	- Irradiation of PET-positive mediastinal and cervical lymph nodes with volumetric modulated arc therapy (5 × 1.8 Gy till 54 Gy) - Stereotactic body radiotherapy (SBRT) of the primary tumor with 5 × 7.5 Gy till 60 Gy	- Irradiation of the primary tumor with volumetric modulated arc therapy (5 × 2.12 Gy till 63.60 Gy) and lymph node metastases received 5 × 2.0 Gy till 60.00 Gy and	- Irradiation of the PET-positive primary tumor with volumetric modulated arc therapy (5 × 2.12 Gy till 61.48 Gy) and extended tumor region including mediastinal lymph nodes with 5 × 1.7 Gy till 49 Gy	- Irradiation of the PET-positive primary tumor with volumetric modulated arc therapy (5 × 2.12 Gy till 63.60 Gy) and hilar and mediastinal lymph node region with 5 × 1.7 Gy till 51 Gy	- Irradiation of the PET-positive primary tumor with volumetric modulated arc therapy (5 × 2.12 Gy till 63.60 Gy) and hilar and mediastinal lymph node region with 5 × 1.7 Gy till 51 Gy	- Irradiation of the PET-positive primary tumor with volumetric modulated arc therapy (5 × 2.12 Gy till 63.60 Gy) and extended high-risk region with 5 × 1.7 Gy till 51 Gy
**Systemic treatment**	- Cisplatin/Pemetrexed (5 cycles) - Durvalumab consolidation (7 cycles)	- Cisplatin/Vinorelbin (2 cycles) - Durvalumab consolidation (12 cycles)	- Cisplatin/vinorelbin (2 cycles) - Carboplatin/Vinorelbin (2 cycles) - Durvalumab consolidation (7 cycles)	- Cisplatin/Vinorelbin (2 cycles) - Durvalumab consolidation (2 cycles)	- Cisplatin/Vinorelbin/Nivolumab (4 cycles) - Nivolumab consolidation (4 cycles)	- Cisplatin/Vinorelbin (2 cycles) - Nivolumab consolidation (14 cycles)
**Relapse or death after treatment**	No	yes	no	Yes	yes	No
**DFS (months)**	31.1	15.6	16.3	11.2	28.7	27.4

## Data Availability

No new data were created or analyzed in this study. Data sharing is not applicable to this article.
